# New York Academy of Medicine

**Published:** 1891-06

**Authors:** 


### NEW YORK ACADEMY OF MEDICINE.—SECTION ON ORTHOPEDIC SURGERY.

*Stated Meeting, February 20, 1891.*

Samuel Ketch, M. D., Chairman.

Dr. Royal Whitman exhibited a case of unusually severe genu-valgum, and one of teno-synovitis of the long extensors of the toes. As in the latter case there was a tuberculous ostitis of the elbow, he considered that the lesion of the foot had a similar origin.

Dr. V. P. Gibney said that in a somewhat similar case, where the tuberculous nature of the lesion was proved by microscopical examination, he had moved the tendons freely, divided the annular ligament, and scraped away the diseased tissue.

Dr. John Ridlon exhibited a man, twenty-one years of age, who, as a result of an injury thirteen years before, was unable to supinate the wrist without causing a backward dislocation of the ulna. The apparatus which he had applied consisted of a leather case, with a metal side, which had been fitted so as to accurately grasp the ulna, by molding it over a plaster cast. He expected that after the patient had worn this apparatus constantly for two or three years, the dislocation would be cured.

Dr. A. M. Phelps read a paper upon

### EXCISION OF THE KNEE JOINT.

Dr. A. B. Judson recognized the conservatism of the treatment advocated by Dr. Phelps, whose paper clearly demonstrated the superiority of excision over amputation; but he thought the time had come for the better conservatism that is found in purely orthopedic treatment. Many of the patients would have made good recoveries if excision had given place to mechanical treatment, which puts an operation out of the question when employed from the beginning of the affection.

Dr. Thomas H. Manley raised the question as to whether a more useful limb would not often result from placing it in a more or less flexed position.

Dr. W. R. Townsend said that the worst results he had seen from excision of the knee had been in cases where the straight position of the limb had not been maintained. Such a faulty position was constantly aggravated by the weight of the body.

Dr. RIDLON said that in spite of the excellent results reported in the paper, he was still of the opinion that this operation upon growing children was wholly unjustifiable, and he was convinced that cases which cannot be cured mechanically—viz., those in which there is an extensive osteo-myelitis, were beyond the reach of anything short of amputation. The exceptionally good results obtained by the author, and a few other surgeons, did not argue against the soundness of the general teaching, that this operation should not be advised for such children.

THE PRESIDENT remarked that, as a rule, orthopedic surgeons were confronted with knee-joint disease in growing children, and the cases mentioned in the paper must have been instances of neglect or of improper treatment.

Dr. PHELPS, in closing the discussion on this paper, said that it might be that the cases had not been properly treated, but some of them had been under the care of good orthopedic surgeons, and he had himself treated some of them ; but he must admit that he could not cure many of these cases by mechanical means. He had not yet had an opportunity of dissecting the parts and actually seeing the organized blood clot, but when a thin shell of bone after an operation becomes strong enough to sustain the weight of one hundred and eighty pounds or more, he thought it reasonable to believe that this great increase in strength was due to something more than mere blood clot. It was true that the majority of cases mentioned in this paper were adults, but many of the operations were performed upon children under twelve years of age, and some of his best results had been between the ages of ten and fourteen years. He urged the adoption of this operation in children not under ten years of age, who were suffering from extensive bone disease, and he believed that the results so obtained were both quicker and better, and often saved the patients from amputation. A perusal of German literature would show that his results were not exceptional, and were similar to those obtained by a number of foreign operators.

Dr. SAMUEL LLOYD read a paper on

### LAMNECTOMY FOR POTT'S DISEASE.

In introducing this subject, he took exception to the terms in common use in speaking of the removal of the posterior arches of the vertebræ, and especially to laminectomy, which was a hybrid term, made up of a Latin and Greek word. He proposed to coin a new word, made from the Greek word meaning a lamina or plate, and another Greek word meaning to remove or cut away. He had collected the histories of thirty-nine cases of operation for Pott's disease.

In cases where abscesses were present, he said it was a comparatively safe procedure to explore the bodies of the vertebræ on their anterior surface, because the approach to the diseased foci was rendered easy by the abscess track, which had already pushed the intervening structures out of the way, and in these cases he advocated exploring the cavity with a view to locating the bony disease and eradicating it if possible. The cases he had tabulated showed that the mortality in cases of lamnectomy, as in ordinary cases of Pott's disease, was greater in adults than in children—57 per cent. in the former, and 10 per cent. in the latter. In only twenty-seven cases was the region involved in the disease stated, and of these, twenty-three were dorsal, which, while not affording sufficient data for an authoritative statement of the effect of the region upon the mortality, still bore out the statement made in a former paper on Lamnectomy for Traumatism of the Spine, that the higher the lesion the greater the mortality. The time of the operation after the onset of the disease varied from four months to seven years. These statistics did not show that any time was better than another for operation, and it was impossible to definitely settle upon any time when, as a rule, operation should be undertaken. No surgeon would interfere in any case in which there were other tubercular affections of any extent, complicating the cord lesion. Macewen's statement that marked hectic was a contra-indication to operation, he considered fallacious. The operation should not be undertaken when there was any chance of recovery, but cases where the chances of recovery without operation were very slight, where continued mechanical treatment yielding little or no result, and where at any moment an extension of the lesion might render the patient hopeless, if it did not destroy his life, had better be operated upon. In cases which showed only progression of the disease in spite of all care, and where an arrested degeneration was set up again, threatening the integrity of the cord, operation should be undertaken early. In performing the operation, he preferred to make a single incision, cutting the spine away from the arch, and leaving them attached to one of the flaps, because this method occupied less time, caused less hemorrhage, and did not interfere with the interspinous ligaments.

### FIBRO-SARCOMA OF THE FOOT.

Dr. Gibney presented a foot which had been removed a few days before from a lady, twenty-eight years of age, who, as a result of bruising the foot against a twig, had suffered pain in the sole for seven years. She had been treated by various appliances, and once by incision, but with negative result. When she came under the speaker's care, eighteen months ago, there was aggravated flat-foot, and a fulness in the sole, which was thought to be due to the cicatricial tissue following the exploratory incision. Apparatus gave only temporary relief, and after consulting with Dr. W. T. Bull, an incision was made along the inner side of the foot, when considerable gelatinous material, and some broken-down bone from the vicinity of the cuboid, were evacuated. Three well-known pathologists in this city examined this tissue, and pronounced it a case of fibro-sarcoma. Subsequently, some more of the tissue was sent to one of these gentlemen, who, from the microscopical appearances of the granulation tissue, pronounced it tuberculosis. On laying the foot open, the bone was found to be fairly healthy. The case was of interest on account of its long duration and the pathological findings.

Dr. Gibney also referred to a case of long-standing hip-joint disease occurring in a girl, twelve years of age, who, as a result of the profuse suppuration, developed amyloid disease of the liver, spleen, and kidneys. As the pathologist, Dr. Tuttle, had found in the cheesy matter removed from a small excavation in one of the acetabula, almost a pure culture of tubercle bacilli, he thought it might be interesting to the members to examine the specimen under the microscope.

---

*Stated Meeting, March 20, 1891.*

Samuel Ketch, M. D., *Chairman.*

### A CASE OF LATERAL CURVATURE.

Dr. A. B. Judson presented a patient, a girl of eleven years, in whom there was marked lateral curvature of the spine, although the line of the spinous processes was straight. The curvature in this case was confined to the bodies of the vertebræ, which were displaced toward the left with the usual signs of rotation of the anterior portion of the vertebral column toward the left. The left scapula was raised, and its posterior border projected sharply backward, an obliquity best seen when the shoulders were observed from above. Stooping developed prominence of the ribs on the left side. Palpation showed the diameter of the chest from the right mammary line to the angles of the left ribs, larger than the corresponding dimension of the other side. The case illustrated the important clinical fact that the gravity of lateral curvature is not to be measured by the curve seen in the spinous processes, but rather by recognizing the amount of rotation. The patient had been under observation two weeks, and the deformity was first noticed by the mother last Summer.

Dr. H. L. Taylor said that after the child had become tired by standing in one position, there seemed to be a slight deviation of the spinous processes, and lateral flexion in the dorsal region seemed a little more restricted towards the left.

Dr. Judson replied that it was common for the degree of deformity to vary with rest and fatigue.

### TRAUMATIC SEPARATION OF THE RIGHT PARIETAL AND OCCIPITAL BONES.

Dr. T. Halsted Myers presented a boy, five months old, who had presented nothing abnormal until two months before, when he had fallen on his head. The injury was quickly followed by great swelling, which gradually diminished. There was no paralysis, and no mental change. Examination showed a cleft in the region of the right half of the lambdoid suture, one inch wide and four inches long, through which a fluctuating mass protruded, probably the membranes distended with cerebro-spinal fluid. It became tense as the child cried, transmitted the cerebral impulse, and on pressure, disappeared almost entirely, with corresponding increased prominence of the anterior fontanelle. The posterior border of the parietal bone seemed also to have suffered a green-stick fracture, one-third of an inch from the edge of the fissure.

### ADJUSTED LOCOMOTION IN THE TREATMENT OF THE RECOVERING STAGE OF HIP-JOINT DISEASE.

Dr. Henry Ling Taylor presented a paper on this subject. He said that the tendency of inflammations of the hip-joint was towards recovery, if favorable conditions were provided. In the stage of acute and progressive inflammation, the treatment by position and counter-extension in the line of deformity, counteract muscular spasm, and protect the joint, and when combined for a short time with recumbency, usually afforded prompt relief to the urgent symptoms, and if persisted in, and modified to meet the varying requirements of the case, ushered in the stage of repair and recovery.

The case of a boy, six and a half years old, who had suffered from hip disease for a year and a half, and had worn a short splint for nine months, was cited to illustrate the prompt relief from properly applied extension, which caused cessation from night cries at once, and within a week, improved the appetite, appearance, weight, and deformity, although he had been rapidly losing ground for several months.

The later stages of these cases in their progress towards recovery, present just as definite though different indications for treatment. As pointed out by Dr. C. Fayette Taylor some twenty years ago, the patient is ready for the motions of walking before he is able to bear weight on the joint. It is not usually for the patient's interest to allow him to walk as soon as active symptoms have disappeared, nor to keep his leg suspended passively, or in a stiff splint, for too long a period. His recently diseased and disused joint and its appendages should be trained and developed, and the reparative process stimulated by the systematic and orderly employment of those elements of locomotion that may be made beneficial, while harmful elements are eliminated. In most cases, this can be conveniently done by the use of the jointed supporting splint, or Dows', which takes the weight of the body upon a perineal strap, but allows the patient free motion at the hip, knee, and ankle. In those recovering cases which present a strong tendency to adduction of the thigh, this may be combated by the elimination of adduction in locomotion, and in all positions, by the use of the perineal crutch bearing in the opposite groin, as described to the Section two years ago. The point is to let the patient have the benefit of the local and general tonic effect of walking, without its harmful pressure and strain at a comparatively early period in the treatment, and to adapt locomotion at all times to the needs of each particular patient. We possess the mechanical means of doing this satisfactorily. Patients are so comfortable, and as a rule, progress so satisfactorily under this treatment, that there need be little temptation to discharge them before a cure is accomplished, even if the supporting apparatus is worn for years. Some of the most satisfactory ultimate results have been after the longest periods of treatment.

The plan above outlined has secured greater comfort and freedom of the patient during a large part of the treatment, and, he was convinced, a larger proportion of good recoveries, as evidenced by better position, more motion, more perfect repair, and greater usefulness than is usually observed after the other methods of treatment.

Dr. Judson thought that the apparatus should be removed gradually, the patient being closely observed after each step of relaxation of treatment. Protection from the weight of the body should be the last thing remitted, and the paper had admirably accented the value of the perineal crutch of the Dows' splint in thus protecting the affected and convalescent joint.

Dr. W. R. Townsend thought that orthopedic surgeons were pretty well agreed as to the necessity for a gradual discontinuance of mechanical appliances, but just when this removal of apparatus should be begun was a most perplexing question. Where the acute symptoms returned, even after a considerable interval, it was an indication that the apparatus had been removed prematurely, and not that the case had relapsed. Dows' splint, although an excellent instrument, was too expensive for dispensary practice, and accordingly it was the custom in the out-patient department of the Hospital for the Ruptured and Crippled, to convert the ordinary long extension splint into a "caliper splint," by removing the adhesive plasters, and fastening the splint to the shoe, so that it may be used as an outside crutch. Flexion at the knee is next allowed, and this, by developing the quadriceps muscle, results in rapid and marked improvement. After an interval of from three to six months, the apparatus is removed, and the patient allowed to go about with a cane for several weeks.

Dr. A. M. Phelps said that the first step in the management of these cases was the treatment of the deformity, and if this were overcome at the outset, relapses were very infrequent. He was of the opinion that patients who were allowed to walk upon the old-fashioned hip splint, experienced an increase in the deformity, and therefore he advocated the use of a very high shoe, with crutches, associated with absolute immobilization of the hip joint. Fixation was not secured by the long traction splint, or any other splint which did not pass up beyond the hip joint. The first essential for successful treatment of an inflamed hip joint was rest. Contrary to the teaching of some, among others Dr. L. A. Sayre, motion did not prevent anchylosis ; in fact, it sometimes caused it. Regarding the removal of apparatus, his rule was to re-apply the apparatus if the motion became more limited, but to discontinue its use if motion increased.

Dr. Royal Whitman remarked that the great disadvantage of treatment with crutches was, that they were abandoned without the advice of the attending surgeon, and on this account he thought that the ordinary traction splint was the best compromise that could be made, especially for dispensary practice.

The Chairman said that in the matter of the removal of apparatus, each case must be a law unto itself. The Dows' instrument had proved very successful in his hands, but some years ago he had been in the habit of employing the old traction splint, by stitching the buckles to the shoe. The disadvantage of this arrangement was that sometimes it produced traumatism. More recently he had made use of a modified Dows', with perineal band, perineal straps, and snap-joint at the knee, and so adjusted within the shoe that a certain amount of traction could be secured without the use of adhesive plaster.

Dr. Taylor, in closing the discussion, said that he did not claim that the long traction splint gave perfect fixation of the joint, but as he believed that counter-traction was practically more important than positive fixation, he favored the former method in the acute stage. He agreed with Dr. Phelps on the necessity for overcoming deformity at the beginning of treatment, and this could usually be done by physiological methods. When to discontinue all treatment, was a matter for individual judgment ; if motion increased after the patient's discharge, it was a good indication that the step had not been taken too early. The treatment outlined by Dr. Townsend was excellent, and apparently the working out of an idea similar to that set forth in the paper. The point to be emphasized was, that it was not necessary to deprive the patient entirely of locomotion until he was completely cured, but that by selecting those elements of locomotion suitable for him, he might enjoy the tonic effects of walking, with due protection to the joint, during the greater part of the treatment, and this protected walking should be continued a long time, if necessary.

In answer to a question as to when he would ordinarily apply the Dows' instrument in very young children, Dr. Taylor said that he thought the average time was about six months of treatment with the traction splint.

### A NEW AUTOMATIC TRACTION HIP SPLINT.

Dr. T. Halsted Myers presented such an instrument.

It consisted of the ordinary long traction hip splint, made with a short sheath and long extension bar, with the addition of a second sliding foot-piece, to which the leg plasters are attached, and of an adjustable spring to exert the traction.

The second foot-piece slides on the extension bar only. Around and above this is a spring, about eight inches long, whose pushing power is regulated at will by a movable circular band, which can be fastened to any part of the extension bar above the spring, by means of a screw.

To adjust the splint, the band above the spring is moved towards the foot-pieces, (which at first lie close together, one on the other,) till the spring is contracted to the desired extent, and then fastened there. There is now a downward pressure on the sliding foot-piece, of the desired amount. The splint is next applied to the patient, the upper foot-piece buckled to the plasters, so as to touch the sole of the shoe. The extension bar is now keyed out, carrying the lower foot-piece away from the upper one; three-quarters of an inch. This also carries the band above the spring down, and increases the power of thes pring somewhat—just how much depending upon its length, the distance between its coils, and the weight of the wire.

When the weight of the body is carried by the splint, any tendency for the straps to bag is overcome by the downward traction exerted by the uncoiling spring, and thus a more equable traction is exerted than has heretofore been possible.

The Chairman referred to a somewhat similar spring apparatus which had been devised by Dr. N. M. Shaffer. At the first meeting of the American Orthopedic Association, the speaker had exhibited a very simple arrangement which he had devised. It consisted in using a strong piece of band rubber, buckled into the traction straps, instead of the usual leather straps. He had found its action satisfactory.

Dr. Judson said that the relaxation of the straps at the foot of the hip-splint had been frequently discussed. He thought it was best obviated by providing an apparatus as nearly inflexible as possible. Also, if the pelvic band is worn at a high level, the strap, being long and deeply curved, allows the patient's body to descend when he stands, further than it would if the pelvic band was at a low level, and the strap crossed it in a direction more nearly horizontal.

But it is questionable whether the relaxation of the straps is of serious moment, because, when the patient stands, the traction made by the weight of the leg takes place of the traction previously made by the adhesive plasters when the patient was recumbent, and is practically much greater and more equable and comfortable.

The joint may be "pumped" when the patient walks without any apparatus, because it is subjected to, and relieved from, the weight of the body above, and the weight of the limb below, at every step. Or, pumping may occur when the patient, wearing the splint, repeatedly lies down and stands up, because then there is an alternation between the great weight of the limb, which produces traction when the patient stands, and the force of mechanical traction, which is comparatively small, and takes effect when the patient is recumbent. The only pumping which the joint gets when the patient walks with the hip-splint, is that which comes from the recurrence and removal of the weight of the splint attached to the limb by adhesive plasters. This interference with the repose of the joint should be conveniently prevented by suspending the splint from the opposite shoulder by properly adjusted webbing.

Dr. Townsend said that the "pumping" action to which the previous speaker had alluded, was one of the great objections urged against the long traction splint. He was much pleased with the new splint which Dr. Myers had presented, but he thought there would be much less heard of this sagging of the straps if attention were paid to the adjustment of the splint in the erect as well as in the recumbent position.

Dr. Taylor agreed with Dr. Judson as to the part played by the splint and band in causing this sagging, and therefore, if the band were discarded, and in its place some substitute like the one which had just been exhibited by him in connection with the Dows' instrument, part of this trouble would be obviated. The traction produced by the weight of the leg is usually sufficient, and hence the traction splint becomes a crutch during locomotion, and an extension splint when the patient is sitting or lying. One of the chief advantages of this splint is that it protects the joint in all positions.

